# A novel single variant in the *MEFV* gene causing Mediterranean fever and Behçet’s disease: a case report 

**DOI:** 10.1186/s13256-017-1552-4

**Published:** 2018-03-01

**Authors:** Maria Zerkaoui, Fatima Zahra Laarabi, Yousra Ajhoun, Bouchra Chkirate, Abdelaziz Sefiani

**Affiliations:** 1 Human Genomic Centre, Faculty of Medicine and Pharmacy, Mohammed V University, Rabat, Morocco; 2 Department of Medical Genetics, National Institute of Health, Rabat, Morocco; 3 Department of Ophthalmology, Mohammed V Military Teaching Hospital, Mohammed V University, Rabat, Morocco; 4 Pediatric Department IV, Children’s Hospital, University Mohammed V, Rabat, Morocco

**Keywords:** *MEFV* gene, Familial Mediterranean fever, Behçet’s disease, FMF-BD coexistence, Case report

## Abstract

**Background:**

Familial Mediterranean fever is an autoinflammatory disease of unknown etiology, characterized clinically by recurrent attacks of sudden-onset fever with arthralgia and/or thoracoabdominal pain and pathogenetically by autosomal recessive inheritance due to a mutation in the *MEFV* gene. Behçet’s disease is an inflammatory disease characterized by recurrent oral and genital aphthous ulcerations, uveitis, and skin lesions. Preliminarily, our literature review suggested that patients with familial Mediterranean fever who also have Behçet’s disease have only a single mutated familial Mediterranean fever gene. The *MEFV* gene mutation responsible for familial Mediterranean fever is probably a susceptibility factor for Behçet’s disease, particularly for patients with vascular involvement, and both disorders can occur concurrently in a patient, as in the present case.

**Case presentation:**

A 10-year-old girl of Moroccan origin presented to our institution for genetic consultation for genetic testing of the *MEFV* gene. She had fever associated with abdominal and diffuse joint pain in addition to headache. These symptoms have oriented pediatricians to familial Mediterranean fever. The evolution was marked by Behçet’s syndrome symptoms. Sanger sequencing followed by complete exome sequencing analysis of the *MEFV* gene for the proband mutation revealed a novel variant. We conclude that the novel single variant c.2078 T > A (p.Met693Lys) could be responsible for the association of familial Mediterranean fever and Behçet’s disease.

**Conclusion:**

To the best of our knowledge, this is the first report of a new variant in exon 10 of the *MEFV* gene in a Moroccan family. This novel variant should be listed in the *MEFV* sequence variant databases.

## Background

Familial Mediterranean fever (FMF) is an ethnically restricted autosomal recessive disease, usually caused by loss-of-function mutations in the *MEFV* gene on the short arm of chromosome 16 [1]. It is the most common genetic disease in the Eastern Mediterranean Basin, affecting in particular Armenians, Turks, North Africans, Ashkenazi Jews, and Arabs. FMF is characterized by recurrent episodes of fever accompanied by peritonitis, pleuritis, arthritis, or erysipelas-like erythema [2]. The most severe complication is the development of renal amyloidosis, which can be prevented by the daily and lifelong administration of colchicine therapy [3]. Behçet’s disease (BD) is a complex chronic and relapsing inflammatory disorder extensively described in young adults from East Asian and Mediterranean countries. Its essential manifestations are oral and genital ulcerations, folliculitis, erythema nodosum, and uveitis. The presence of vasculitis worsens the prognosis, with the potential to introduce life-threatening complications such as thrombophlebitis, arterial aneurysms, and occlusion [4]. The etiology of BD is unknown. A higher frequency of *MEFV* mutations in patients with BD with respect to their ethnicity has previously been demonstrated [5]. Other groups have confirmed these data and found an association between the presence of *MEFV* mutations and the severity of vasculitis.

The *MEFV* gene encodes the pyrin/marenostrin expressed primarily in the myeloid cell lineage, which modulates cell susceptibility to apoptosis through the regulation of inflammation [6]. More than 300 variants throughout the *MEFV* gene have been identified, with M694V, M680I, and E148Q being the most common [7].

We report a case of a Moroccan patient with FMF who also had BD (FMF-BD) who had been regarded as having FMF alone 3 years from the onset of disease. Molecular testing revealed a novel single variant that could be responsible for the association of FMF and BD.

## Case presentation

**Fig. 1:**
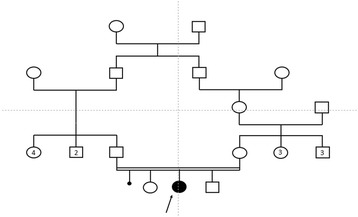
Pedigree of the patient’s family

**Fig. 2:**
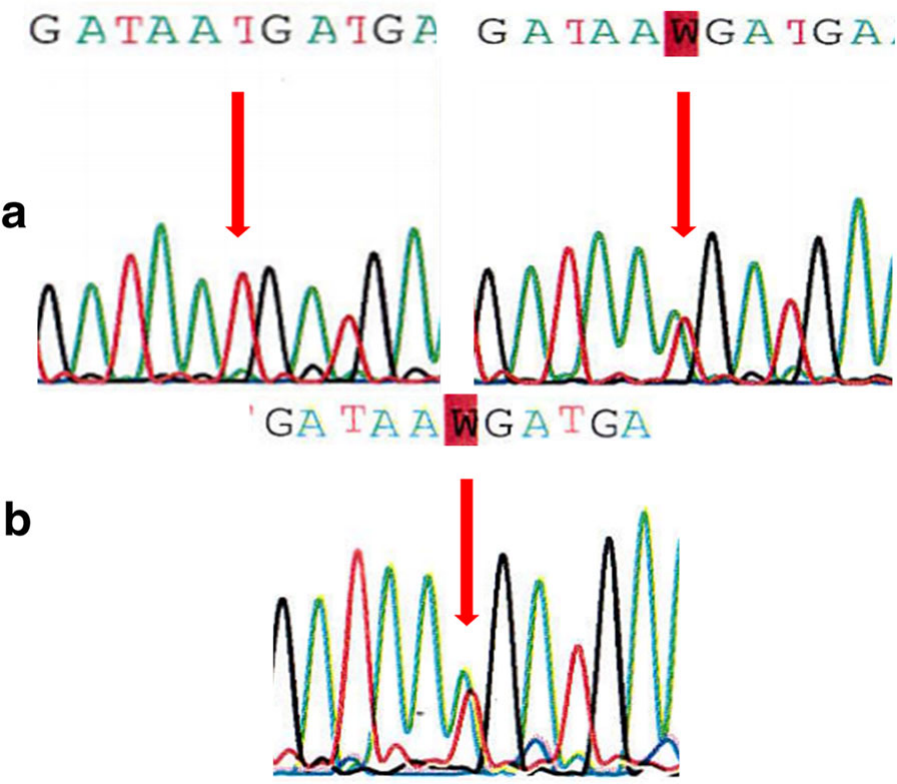
Electrophoregrams of the novel heterozygous missense mutation at the 2078 nucleotide position of the *MEFV* gene. **a** (red arrow) Normal sequence of unaffected mother and heterozygous mutation c.2078 T > A (p.Met693Lys) of unaffected father. **b** (red arrow) Heterozygous mutation c.2078 T > A (p.Met693Lys) of our patient, a Moroccan girl presenting with coexistence of familial Mediterranean fever and Behçet’s disease

In 2015, we received in genetic consultation a consanguineous 10-year-old Moroccan girl, the second of three siblings, for genetic testing of the *MEFV* gene (Fig. 1). At admission, the patient had a periodic fever with abdominal pain, mucocutaneous symptoms (pseudo erysipelas), vomitus, and joint pain (monoarthritis) in addition to headache. Pediatricians had excluded acute rheumatic fever on the basis of normal levels of antistreptolysin O and normal echocardiographic results. During the treatment, the patient demonstrated an intolerance to colchicine with diarrhea and abdominal pain, so the response to colchicine was not determined. We performed deoxyribonucleic acid (DNA) analysis. Initially, we excluded recurrent mutations in exons 2 and 10. Molecular testing of the entire *MEFV* gene revealed the novel variant c.2078 T > A, p.M693K, predicted as damaging and deleterious by the SIFT and PROVEAN tools (Fig. 2). This result pointed to a diagnosis of FMF.

Meanwhile, the child’s condition had worsened 2 weeks after her admission; high fever had persisted through the prior week and was associated with intense headache and abdominal pain that were relieved with opioids. This severity is atypical in FMF. The laboratory data revealed an elevated level of C-reactive protein, although the levels of white blood cells were within normal range and antiphospholipid antibodies were negative. Cerebral angiography showed cerebral venous thrombosis of the junction and the sigmoid sinus on the left and right sides. The patient’s clinical history had been taken over. For a few months, the patient was showing one episode of aphthosis in the buccal mucosa as well as urogenital ulcers. She was given a bolus of intravenous methylprednisolone for 3 consecutive days, followed by oral prednisone; simultaneously, we administered intravenous anticoagulation (enoxaparin sodium) followed by oral acenocoumarol to maintain an international normalized ratio between 2 and 3. Resolution of the thrombus was achieved by the time of the 6-month follow-up visit. Thrombophlebitis of the left peroneal vein visualized by Doppler echography appeared 2 weeks after starting medical treatment. An ophthalmologic examination revealed papillary edema degree 2 with scleromalacia. We reconsidered the diagnosis according to the established Tel-Hashomer clinical criteria and the International Study Group for Behçet Disease classification criteria for diagnosis of BD [8]. According to these criteria, the young girl had FMF-BD. We completed the genetic analysis of her parents. Her mother was normal, and her father was heterozygous for the same mutation but remained asymptomatic.

## Discussion

FMF is a genetic disease characterized by attacks of fever, arthritis, serositis, and abdominal pain. BD is an inflammatory disorder with a genetic basis that is characterized by oral and genital ulcers, uveitis, pustular erythematous cutaneous lesions, arthritis, central nervous system involvement, and possible vascular manifestations such as venous thrombosis, arteritis, and aneurysms. The diagnosis of BD is independently associated with higher incidence of FMF, especially in females, among people of Arab descent. Watad *et al*.’s data implied that understating the differentiation between FMF and BD was not evident and clear in a real-life population of patients with BD [9]. The clinical picture of FMF is often similar to that of BD with regard to inflammatory reactions with activation of neutrophils and therapeutic efficacy of colchicine, and the possible coexistence of the former should be considered, particularly in cases with periodic fever as in our patient. Because *MEFV* encodes the neutrophil protein pyrin, mutations in the *MEFV* gene may play an important role in the pathogenesis of BD [10]. One hypothesis is that a reduced level of functional pyrin on the one hand, presumably characterizing heterozygosity, and increased demand for pyrin on the other hand, by activated granulocytes of BD, may ultimately lead to FMF attacks [11].

Diagnosis of FMF remains clinical and requires information about family history and response to colchicine. Our patient presented with FMF symptoms of fever, abdominal pain, joint pain, and erysipelas-like erythema. In the literature, severe abdominal and chest pain are reported to be the most typical symptoms, occurring in more than 90% and more than 40% of patients, respectively. Suggestive of an FMF diagnosis is the presence of a recurrent erysipelas-like rash on the skin of the inferior limbs [12]. Our patient presented with a recurrent headache, which is an atypical symptom of FMF. She kept having headaches, but her pediatricians considered this symptom a consequence of fever. She presented with aphthosis in the buccal and genital regions, and after investigation, we objectified cerebral and peroneal vein thrombophlebitis, symptoms related to BD. It has been reported that the *MEFV* genetic mutation responsible for FMF is probably a susceptibility factor for BD, particularly in cases with vascular involvement, and that both disorders can coexist in a patient, as was the case in our patient [10]. *MEFV* mutations might be a more crucial genetic factor in female patients with vascular involvement [13].

In 2001, Livneh *et al*. [11] suggested that about 60% of patients with FMF-BD have only a single mutated FMF gene (*MEFV*). Moreover, in three families in that study, mutational analysis revealed at least one heterozygous family member who shared the noncarrier chromosome with the FMF-BD proband but remained unaffected. This may be explained by the presence of a low-penetrance mutation, not detected by that team, in noncoding regions (for example, intron, promoter, enhancer), which causes FMF expression only in a fraction of carriers [11]. A significant number of patients diagnosed with FMF show only a single mutation despite sequencing of the entire *MEFV* genome region or other autoinflammatory genes, and this has led to reconsideration of simple loss of function of the recessive model of FMF inheritance. In some cases, FMF is a dominant condition with low penetrance and variable disease expression, presenting not only in homozygous subjects but also in heterozygous subjects [2].

According to their phenotype classification in the publication by Balci *et al*. [14], patients with a classic phenotype who were genetically confirmed to have *MEFV* mutations are defined as phenotype I. Phenotype III was defined as those who have no clinical signs but present with the required genotype, whereas phenotype II refers to patients who develop amyloid A amyloidosis without any previous attacks typical of FMF [14]. Therefore, our patient’s father was asymptomatic but carried the same novel variant c.2078 T > A in the *MEFV* gene.

FMF is typically inherited in an autosomal recessive fashion, although there have been rare reports of dominant inheritance with specific heterozygous mutations and complex alleles [15]. The parents of our proband were clinically normal. Genetic analysis of the mother’s *MEFV* gene was normal. The father, even if he was unaffected, carried the proband mutation.

There is good evidence that position M694 of the pyrin molecule is critically important for its normal function. The p.M694 is located in the putative binding site of caspase-1, and the substitution or deletion of this residue may interfere with the inhibitory interaction between pyrin and caspase-1 and thus promote interleukin-1β generation [16]. This reasoning could be applied to the p.M693K mutation of the pyrin molecule found in our patient and thus highly suggests its pathogenic effect. Our patient carried a single mutated allele of the *MEFV* gene that could be responsible for the coexistence of FMF and BD.

## Conclusions

We present what is, to the best of our knowledge, the first identification of an *MEFV* mutation in a Moroccan family with associated FMF and BD. To the best of our knowledge, this mutation has not been reported before. Therefore, we think that the c.2078 T > A (p.M693K) mutation should be listed in the *MEFV* sequence variants of FMF-BD databases and may help to increase public awareness of this rare association in the Moroccan population. To identify specific phenotype/genotype correlations exactly, the novel mutation and its relationship should be confirmed by studying homozygous individuals for this mutation.

## Abbreviations

BD: Behçet’s disease
FMF: Familial Mediterranean fever
FMF-BD: Familial Mediterranean fever with associated Behçet’s disease
